# Sulfhydryl Sulfobetaine Stabilized Palladium Nanoparticles with High Peroxidase-like Activity for Enhanced Glutathione Detection and Tumor Suppression

**DOI:** 10.3390/biom16071003

**Published:** 2026-07-09

**Authors:** Ruyu Li, Yanshuai Cui, Shukai Li, Xianbing Ji, Longgang Wang

**Affiliations:** 1 Hebei Key Laboratory of Nano-Biotechnology, Hebei Key Laboratory of Applied Chemistry, College of Environmental and Chemical Engineering, Yanshan University, Qinhuangdao 066004, China; liruyuys@stumail.ysu.edu.cn; 2 State Key Laboratory of Materials Science and Technology, Yanshan University, Qinhuangdao 066004, China; 3 Hebei Key Laboratory of Agroecological Safety, Department of Environmental Engineering, Hebei University of Environmental Engineering, Qinhuangdao 066102, China; cuiyanshuai@hebuee.edu.cn (Y.C.); jixianbing@hebuee.edu.cn (X.J.); 4 College of Materials Science and Chemical Engineering, Harbin Engineering University, Harbin 150001, China; lishukai1998@hrbeu.edu.cn

**Keywords:** palladium, sulfhydryl sulfobetaine, glutathione, detection, photothermal therapy

## Abstract

Glutathione (GSH)-responsive nanozymes have attracted increasing attention for biosensing and cancer therapy. However, their practical applications are often limited by aggregation and insufficient catalytic activity. Herein, we report a zwitterionic sulfobetaine-modified palladium nanozyme (SH-SB/Pd NPs) that addresses these limitations by integrating high catalytic activity. The zwitterionic ligand simultaneously stabilizes Pd nanoparticles and preserves accessible catalytic sites, resulting in markedly enhanced peroxidase-like activity. SH-SB/Pd NPs efficiently catalyze H_2_O_2_ decomposition to generate multiple reactive oxygen species (^1^O_2_, O_2_^•−^ and •OH), enabling favorable affinity for TMB (*K*_*m*(*TMB*)_ = 0.28) and sensitive colorimetric GSH detection with a low detection limit of 0.135 μM. Benefiting from their long-term antifouling properties and ROS-generating capability, SH-SB/Pd NPs also exhibit potent antitumor activity, achieving 76.56% inhibition of HeLa cells under 808 nm laser irradiation. This work establishes a zwitterionic nanozyme platform that improves catalytic activity, stability, and therapeutic performance, offering a promising strategy for biosensing and synergistic cancer therapy.

## 1. Introduction

As a cellular antioxidant, free radical scavenger and detoxification agent, glutathione (GSH) is essential for carcinogen removal and detoxification [1,2]. Cancer treatment has gradually shifted from traditional single-modality therapy to personalized and precision combination therapy [3]. In cancer cells, elevated GSH levels are associated with tumor progression and increased resistance to chemotherapy drugs [4]. Therefore, GSH detection is important for health assessment, cancer prevention, and diagnosis. Compared with easily quenched fluorescence spectrometry [5], high-cost high-performance liquid chromatography (HPLC) [6], electrochemical methods [7] with long testing times and other analytical methods with complex operations, the colorimetric method [8] has the advantages of being intuitive, economical and rapid. In the process of enzyme-based colorimetric detection of GSH, high-performance enzymes are important for the establishment of a reliable and sensitive GSH bioassay platform [9].

Although glucose oxidase (GOD) and horseradish peroxidase (HRP) have long been used in colorimetry, deactivation caused by environmental factors and complex extraction processes have limited their application [10,11]. Since 2007, Fe_3_O_4_ NPs [12] have been reported to exhibit peroxidase-like activity, monometallic nanoparticles, metal alloys [13,14], metal oxides [15,16] and other hybrid nanomaterials such as carbon nanomaterials [17] and metal–organic framework materials [18] have also become common materials for the preparation of nano-enzymes. As an important member, noble metal nano-enzymes have the advantages of simple preparation, high specific surface area, adjustable activity, and convenient storage compared with natural enzymes, such as Ag NPs [19], Au NPs [20], Pt NPs [21] and Pd NPs [22]. Pd NPs have excellent enzyme-like properties and electronic and optical properties and have been applied in the construction of GSH biological detection platforms and photothermal therapy treatment (PTT) of tumors [23,24]. However, Pd NPs obtained through direct reduction tend to precipitate easily in aqueous solution, making it difficult to effectively promote the colorimetric reaction.

The surface properties of Pd NPs were improved by ligand modification, so the stability and catalytic performance of Pd NPs in solution were improved. For example, poly (dienyl dimethyl ammonium chloride) (PDDA)-modified Pt and Pd NPs prepared by You et al. [25] exhibited excellent stability over a wide pH range compared with citrate capped Pt and Pd NPs, and they have been applied to catalytic hydrogenation of 4-nitrophenol in the presence of NaBH_4_. Palladium nanoparticles prepared by Mahmoud Nasrollahzadeh et al. [26] using a new Schiff base modified chitosan-kaolin (Pd NPs@CS-Kao) as a carrier showed good catalytic performance and effective antibacterial properties against *Escherichia coli*. Highly crystalline spherical 7–9 nm acid lemon tree gum loaded palladium nanoparticles (LAG-Pd NPs) synthesized by Kondaiah Seku et al. [27] have peroxidase-like activity and are used for colorimetric detection of H_2_O_2_ and glucose. Compared with traditional organic molecules, proteins, plant extracts, metal–organic frameworks, carbon nanomaterials and other materials, the zwitterionic materials (such as poly (EK) peptides and TMAP) form a strong hydration layer due to their balanced charges, which helps reduce nonspecific protein adsorption and improves colloidal stability in biological environments [28].

Sulfhydryl sulfobetaine (SH-SB) is a zwitterionic material that contains both a sulfonic acid group and an amino group in one molecular chain. The sulfhydryl group of SH-SB binds to Pd NPs to optimize the surface charge structure of Pd NPs and regulate their stability and catalytic activity. Herein, 7.3 ± 2.4 nm SH-SB/Pd NPs uniformly dispersed in aqueous solution were prepared by SH-SB modification of Pd NPs in this work. Compared with modifications using elm pod polysaccharide (EPP), daptomycin (Dap), and cysteine (Cys), SH-SB modification can expose more catalytic active sites of Pd NPs, and amphiphilic groups of SH-SB/Pd NPs can participate in electron transfer, synergistically enhancing the catalytic efficiency of the enzyme-like reaction. The catalytic mechanism of SH-SB/Pd NPs on TMB was investigated by active oxygen (ROS) inhibitors. SH-SB/Pd NPs exhibited high affinity toward TMB through an enzyme kinetics study. Based on the good peroxidase-like activity of SH-SB/Pd NPs, a sensitive and reliable colorimetric detection method for GSH was established (0.135–180 μM, LOD = 0.135 μM). Finally, SH-SB/Pd NPs showed a good inhibitory effect on HeLa cells in vitro depending on their good photothermal properties. SH-SB/Pd NPs have broad application prospects in the biomedical field for their good stability, catalytic activity and tumor inhibitory ability.

## 2. Materials and Methods

### 2.1. Materials

Hydrogen peroxide (H_2_O_2_, 30%), sodium tetrachloropalladate (Na_2_PdCl_4_, 98%), 3,3′,5,5′-tetramethylbenzidine (TMB, 99%), glutathione (GSH, 98%), o-phenylenediamine (OPD, 98%), thiazol blue (MTT, 98%), 2,2′-azino-bis(3-ethylbenzothiazoline-6-sulfonic acid) (ABTS, 98%), and sodium borohydride (NaBH_4_, 98%) were purchased from Aladdin (Shanghai, China). Dialysis kits with a molecular weight cut-off (MWCO) of 8000–14,000 Da were obtained from Spectrum Laboratories Inc. (Shanghai, China). Sulfhydryl sulfobetaine (SH-SB) was prepared by referring to the previously reported methods [29]. All chemicals were of analytical grade and used as received.

### 2.2. Preparation and Characterization of SH-SB/Pd NPs

Pd nanoparticles (Pd NPs) were synthesized by adding 600 μL of NaBH_4_ solution (40 mM) into 4 mL of Na_2_PdCl_4_ solution (2 mM) under stirring. Subsequently, 40 μL of the SH-SB solution (5 mg/mL) was rapidly added. The mixture was purged with nitrogen (N_2_) for 0.5 h and then incubated at 30 °C for 3 h. The product was purified by dialysis for 24 h.

UV-Vis spectra were recorded using a UV-visible spectrophotometer (UV-TU1810, Beijing Puxi General Instrument CO., Ltd. (Beijing, China)). Zeta potential and hydrodynamic size were measured using a laser particle size analyzer (Zetasizer Nano-ZS90, Malvern Panalytical Ltd. (Worcestershire, UK). The morphology of SH-SB/Pd NPs was characterized by transmission electron microscopy (HT7700, Hitachi High-Technologies Corporation (Tokyo, Japan)). X-ray diffraction (XRD) patterns were obtained using an X-ray diffractometer (Kratos AXIS Ultra, Shimadzu Corporation (Kyoto, Japan)). X-ray photoelectron spectroscopy (XPS) analysis was performed using the Thermo ESCALAB 250Xi system (Thermo Fisher Scientific (Waltham, MA, USA)). HeLa cells were obtained from a standard cell culture collection center to evaluate the anti-tumor efficacy of SH-SB/Pd NPs. Mouse serum was collected from female KM mice for GSH detection.

### 2.3. Study on the Enzyme-like Activity of SH-SB/Pd NPs

To evaluate oxidase-like activity, 50 μL of SH-SB/Pd NPs solution (C_Pd_ = 1.725 mM) was added to a mixture containing 1 mL of TMB solution (0.6 mM) and 200 μL of NaAc-HAc buffer (0.2 M, pH = 4). The reaction mixture was incubated for 5 min at 30 °C and 600 rpm. The UV-Vis absorption spectra were then recorded in the range of 500–800 nm.

For peroxidase-like activity assessment, 50 μL of SH-SB/Pd NPs solution (C_Pd_ = 1.725 mM) was mixed with 1 mL of TMB solution (0.6 mM) and 200 μL of NaAc-HAc buffer (0.2 M, pH = 4). After incubation for 5 min, 50 μL of H_2_O_2_ solution was added, followed by further incubation under identical conditions. The absorbance spectrum (500–800 nm) was then measured. The same procedures were used to evaluate catalytic activity using OPD and ABTS as substrates. Specifically, TMB (0.6 mM) was replaced with OPD (5 mM) or ABTS (2.5 mM), respectively.

### 2.4. Exploration of Environmental Effect and Enzyme Kinetics

In total, 200 μL NaAc-HAc solution (0.2 M, pH = 1–8) and 1 mL TMB solution (0.6 mM, pH = 1–8) were mixed and incubated for 5 min. Then, 50 μL of SH-SB/Pd NPs solution (C_Pd_ = 1.725 mM) was added into the mixture and incubated at 600 rpm at different temperatures (T = 10–80 °C) for 5 min, the absorbance of 500–800 nm was determined. The optimal reaction conditions of SH-SB/Pd NPs were obtained. After incubation of 50 μL SH-SB/Pd NPs solution (C_Pd_ = 1.725 mM) and 200 μL NaAc-HAc buffer solution (pH = 1–8) at different temperatures (T = 10–80 °C) for 2 h, the enzyme-like activity was determined using TMB as substrate. The catalytic stability of SH-SB/Pd NPs was obtained.

The catalytic kinetic parameters of SH-SB/Pd nanoparticles were calculated using different concentrations of H_2_O_2_ and TMB as substrate. In total, 50 μL SH-SB/Pd NPs solution (C_Pd_ = 1.725 mM), 200 μL NaAc-HAc buffer solution (0.2 M, pH = 4), 50 μL H_2_O_2_ solution (0.1 M) and 1 mL TMB solution (0.05–0.6 mM) were mixed and incubated at 40 °C for 5 min. Then, time scanning was performed at 652 nm. To find the effect of H_2_O_2_, 50 μL H_2_O_2_ solution (0.5–10 mM) and 50 μL SH-SB/Pd NPs solution (C_Pd_ = 1.725 mM) were added to the mixture of 1 mL TMB solution (0.6 mM) and 200 μL NaAc-HAc buffer solution (0.2 M, pH = 4). Time scanning was performed at 652 nm.

### 2.5. Study on the Catalytic Mechanism of SH-SB/Pd NPs

NaN_3_, BQ, EDTA and IPA solutions (50 mM) were used as ^1^O_2_, O_2_^•−^, h^+^ and •OH inhibitors, respectively. A total of 1 mL TMB solution (0.6 mM), 50 μL SH-SB/Pd NPs solution (C_Pd_ = 1.725 mM) and 200 μL NaAc-HAc buffer solution (0.2 M, pH = 4) were added into 2 mL EP tubes. Then 100 μL NaN_3_, BQ, EDTA or IPA solution (50 mM) was added to the mixture and incubated for 3 min. The catalytic mechanism of SH-SB/Pd NPs as an oxidase-like enzyme was investigated by analyzing the UV–vis absorption spectra (500–800 nm).

In total, 200 μL NaAc-HAc buffer solution (0.2 M, pH = 4), 50 μL SH-SB/Pd NPs (C_Pd_ = 1.725 mM), 50 μL H_2_O_2_ solution (0.1 M) and 100 μL NaN_3_, BQ, EDTA or IPA solution (50 mM) were incubated in 2 mL EP tubes for 20 min. Then, 1 mL of TMB solution (0.6 mM) was added and the solutions were incubated for 3 min. The catalytic mechanism of SH-SB/Pd NPs as a peroxidase mimic was obtained by comparing the absorbance at 652 nm before and after adding ROS inhibitors.

### 2.6. Construction of a GSH Detection Platform

In total, 50 μL SH-SB/Pd NPs solution (C_Pd_ = 1.725 mM) and 1 mL TMB solution (0.6 mM) were reacted for 5 min. A total of 50 μL H_2_O_2_ solution (20 mM) and 400 μL NaAc-HAc buffer (0.2 M, pH = 4) were added successively and reacted for 5 min. The absorbance of the mixture at 652 nm after the reaction was recorded as A_0_. The buffer solution was replaced by GSH solution of different concentrations (0.2–300 μM, pH = 4), and the absorbance of the solutions at 652 nm after reaction was recorded as A_1_. The absorbance changed by GSH was denoted as ΔA, ΔA = A_0_ − A_1_. With ΔA as the horizontal coordinate and the GSH concentration as the vertical coordinate, the standard curve of the GSH biological detection platform was obtained.

### 2.7. Study on the Photothermal Properties of SH-SB/Pd NPs

In total, 1 mL SH-SB/Pd NPs solution (C_Pd_ = 1.725 mM) was irradiated with an 808 nm laser (η = 1.75 W/cm^2^) in 2 mL EP tubes, and the temperature changes were recorded. In addition, the photothermal properties of SH-SB solution (5 mg/mL), Pd NPs solution (C_Pd_ = 1.725 mM) and Na_2_PdCl_4_ solution (2 mM) were compared. The stability of the photothermal properties was tested by repeated exposure to the 808 nm laser. The photothermal conversion efficiency (η) was calculated according to Equation (1).

(1) $$\eta \;(\%)=\frac{{\mathrm{hs}(T}_{\max}-T_{\mathrm{sur}})-Q_{s}}{I(1-10^{-A_{808}})}\times 100$$

h: Thermal conductivity, s: Outside area, $T_{\max}$: Maximum temperature of samples, $T_{\mathrm{sur}}$: Surrounding temperature, $Q_{s}$: Absorbed heat, I: Laser power, $A_{808}$: Absorbance at 808 nm of samples.

### 2.8. Study on Cytotoxicity of SH-SB/Pd NPs

To investigate the cytotoxicity of SH-SB/Pd NPs, HeLa cells were treated with 200 μL Pd NPs solution (50 μg/mL), SH-SB solution (50 μg/mL) and SH-SB/Pd NPs solution (C_Pd_ = 6.25–100 μg/mL) for 24 h, respectively. Some experimental holes were irradiated by the 808 nm laser (η = 1.75 W/cm^2^) for 10 min. Finally, the above medium was replaced by 100 μL MTT solution (0.5 mg/mL). After 4 h, 150 μL DMSO was added and the absorbance at 492 nm was measured. Cell viability was calculated according to Formula (2).

(2) $$\mathrm{Cell}\;\mathrm{viability}\;(\%)\;=\frac{A_{\mathrm{Samples}}}{A_{\mathrm{Control}}}\times 100$$

$A_{\mathrm{Samples}}$: Absorbance of experimental groups, $A_{\mathrm{Control}}$: Absorbance of the blank group.

### 2.9. Statistical Analysis

Quantitative data were presented as mean ± standard deviation (SD). Significance analysis (* *p* < 0.05, ** *p* < 0.01 and *** *p* < 0.001) was determined by *t*-test (*n* = 3).

## 3. Results and Discussion

### 3.1. Characteristics of SH-SB/Pd NPs

**Figure 1:**
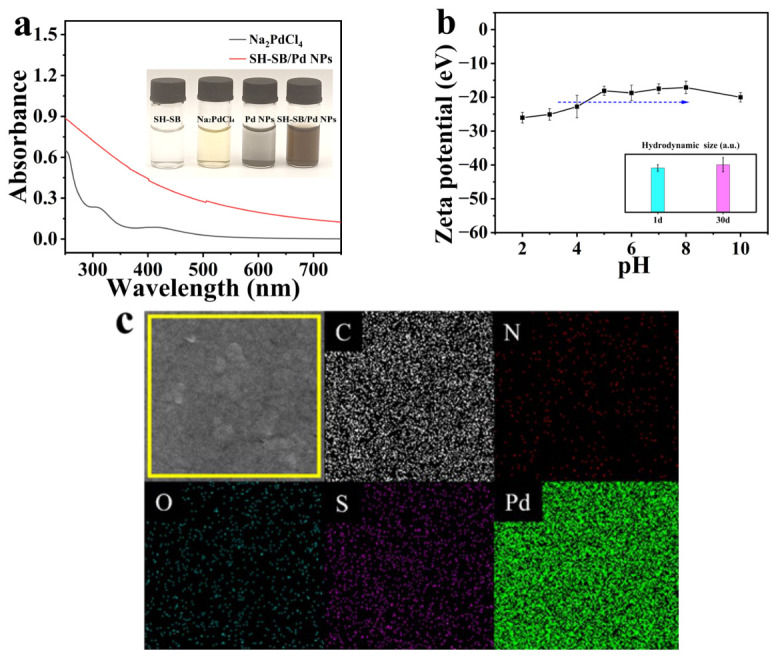
(**a**) UV-Vis absorption curves of Na_2_PdCl_4_ and SH-SB/Pd NPs, (**b**) hydrodynamic size and zeta potential and (**c**) element mapping of SH-SB/Pd NPs.

Figure 1a shows the UV-Vis absorption spectrum of SH-SB/Pd NPs obtained by a UV-Vis spectrophotometer. The metal precursor Na_2_PdCl_4_ exhibited two characteristic absorption peaks at 320 nm (PdCl_4_^2−^) and 420 nm (partially hydrolyzed Pd(II) chlorocomplexes, such as [PdCl_3_(H_2_O)]^−^), as previously reported [30,31]. After reaction, no obvious absorption peaks were observed for SH-SB/Pd NPs. The disappearance of the characteristic absorption peaks of Na_2_PdCl_4_ indicates the complete reduction in Pd^2+^ to Pd^0^. During the reaction process, Pd^2+^ was reduced to Pd^0^ by NaBH_4_ and subsequently stabilized by SH-SB, resulting in the formation of stable SH-SB/Pd NPs.

Zeta potential and hydrodynamic sizes of SH-SB/Pd NPs in NaAc-HAc buffer solution (pH = 2–10) were tested by DLS, as shown in Figure 1b. Under different pH conditions, the zeta potential of SH-SB/Pd NPs ranged from −26.03 eV to −17.1 eV, indicating that pH had little effect on the zeta potential. Zwitterion SH-SB prevented the surface electrochemical properties of SH-SB/Pd NPs from changing. Within 30 days, the hydrodynamic size of SH-SB/Pd NPs changed from 50.06 nm to 53.75 nm. This behavior is likely associated with changes in the hydration layer and electrostatic interactions of the surface zwitterionic ligands, which may contribute to the enhanced enzyme-like activity of SH-SB/Pd NPs.

**Figure 2:**
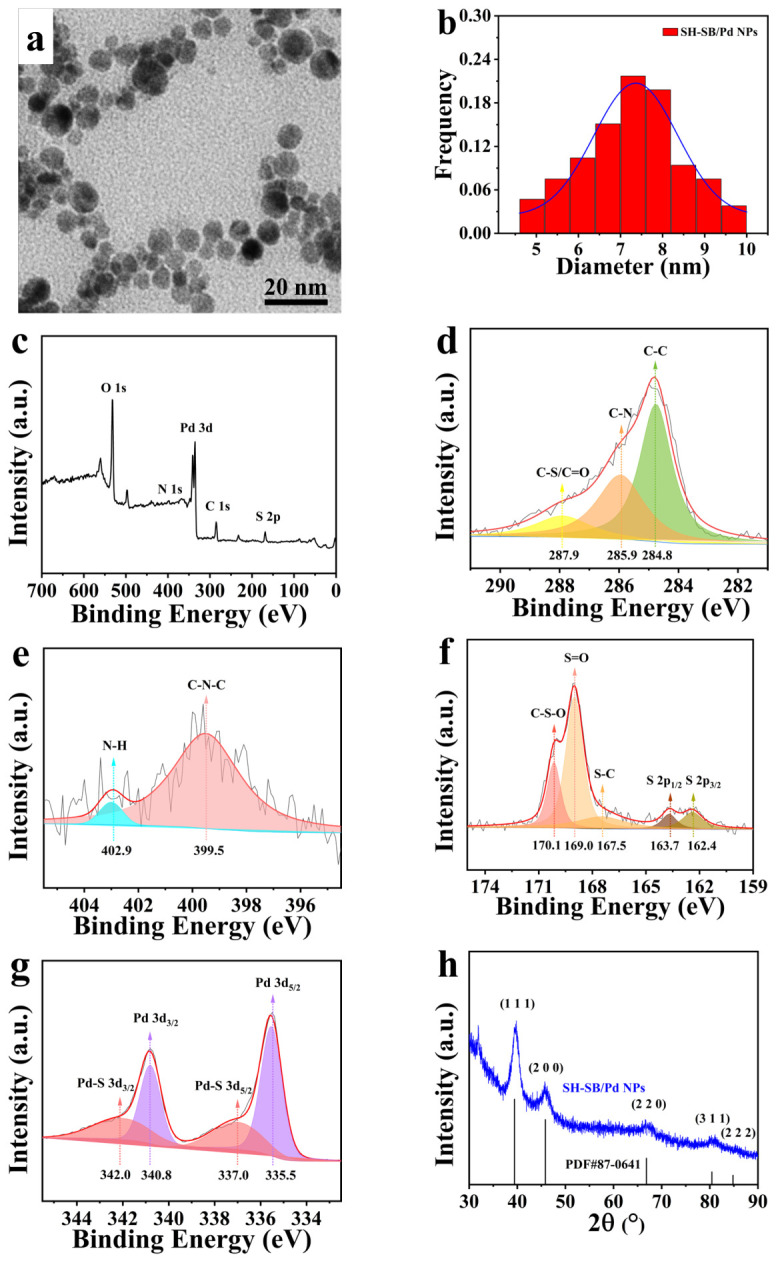
(**a**,**b**) TEM images and statistical analysis, (**c**–**g**) XPS spectra and (**h**) XRD spectra of SH-SB/Pd NPs.

Elemental mapping analysis of SH-SB/Pd NPs (Figure 1c) shows that C, N, O, S, and Pd elements are uniformly distributed throughout the nanoparticles, confirming the successful surface modification of Pd NPs with SH-SB and indicating a homogeneous nanostructure. The particle size of SH-SB/Pd NPs was 7.3 ± 2.4 nm, with a relatively uniform distribution, as shown in Figure 2a,b. The good aqueous stability, excellent dispersibility, and small particle size are expected to be favorable for the enzyme-like activity of SH-SB/Pd NPs.

Figure 2c is the XPS spectrum of SH-SB/Pd NPs, which contains C, N, O, S and Pd elements. In Figure 2d, the absorption peaks at 284.8 eV, 285.9 eV, and 287.9 eV were attributed to the C-C, C-N, and C-S/C=O bonds, respectively. The absorption peaks at 399.5 eV and 402.9 eV in Figure 2e were attributed to the C-N-C and N-H bonds, respectively. In Figure 2f, the absorption peaks at 162.4 eV, 163.7 eV, 167.5 eV, 169.0 eV and 170.1 eV were attributed to the S 2p_3/2_, S 2p_1/2_, S-C, S=O and C-S-O bonds, respectively. In Figure 2g, the absorption peaks at 335.5 eV and 340.8 eV were attributed to Pd 3d_5/2_ and Pd 3d_3/2_, and the absorption peaks at 337.0 eV and 342.0 eV were generated by Pd-S 3d_5/2_ and Pd-S 3d_3/2_, respectively, indicating that after Pd^2+^ was reduced to Pd^0^, part of Pd^0^ with SH-SB to generate Pd-S bond, which enabled SH-SB/Pd NPs existed stably in aqueous solution. The X-ray diffraction peaks at 39.398°, 45.813°, 66.796°, 80.400° and 84.778° in the XRD pattern of SH-SB/Pd NPs corresponded to the (1 1 1), (2 0 0), (2 2 0), (3 1 1) and (2 2 2) crystal planes of Pd, respectively. The lattice parameters were a = b = c, with α = β = γ = 90°, indicating a cubic crystal system. During the reaction, Pd^2+^ was reduced to Pd^0^, and the Pd in SH-SB/Pd NPs adopted a face-centered cubic (fcc) structure [32].

### 3.2. Enzyme-like Activity of SH-SB/Pd NPs

**Figure 3:**
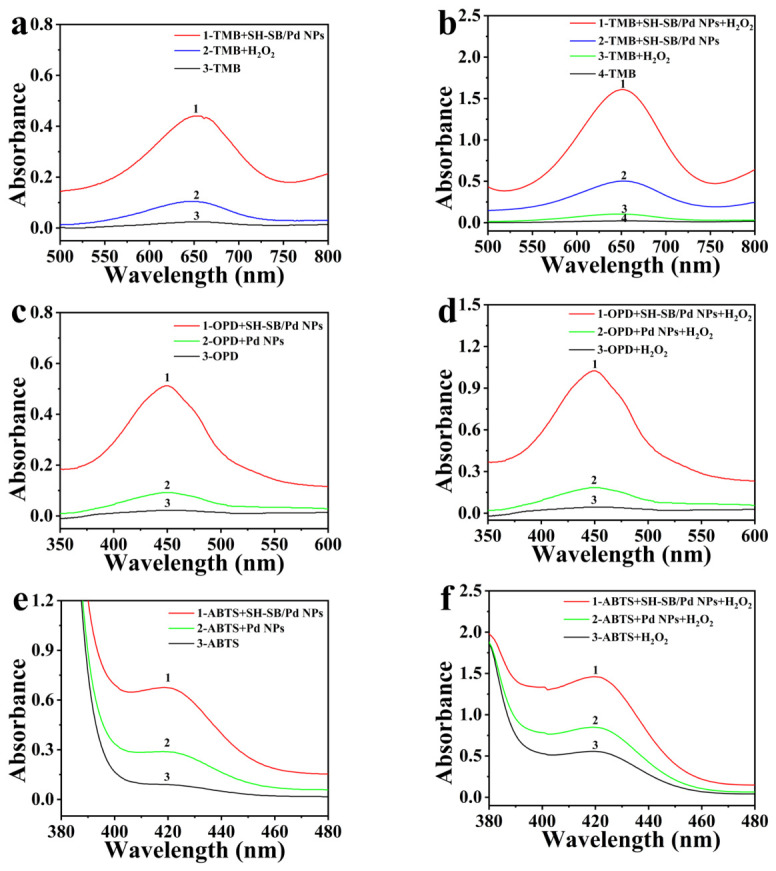

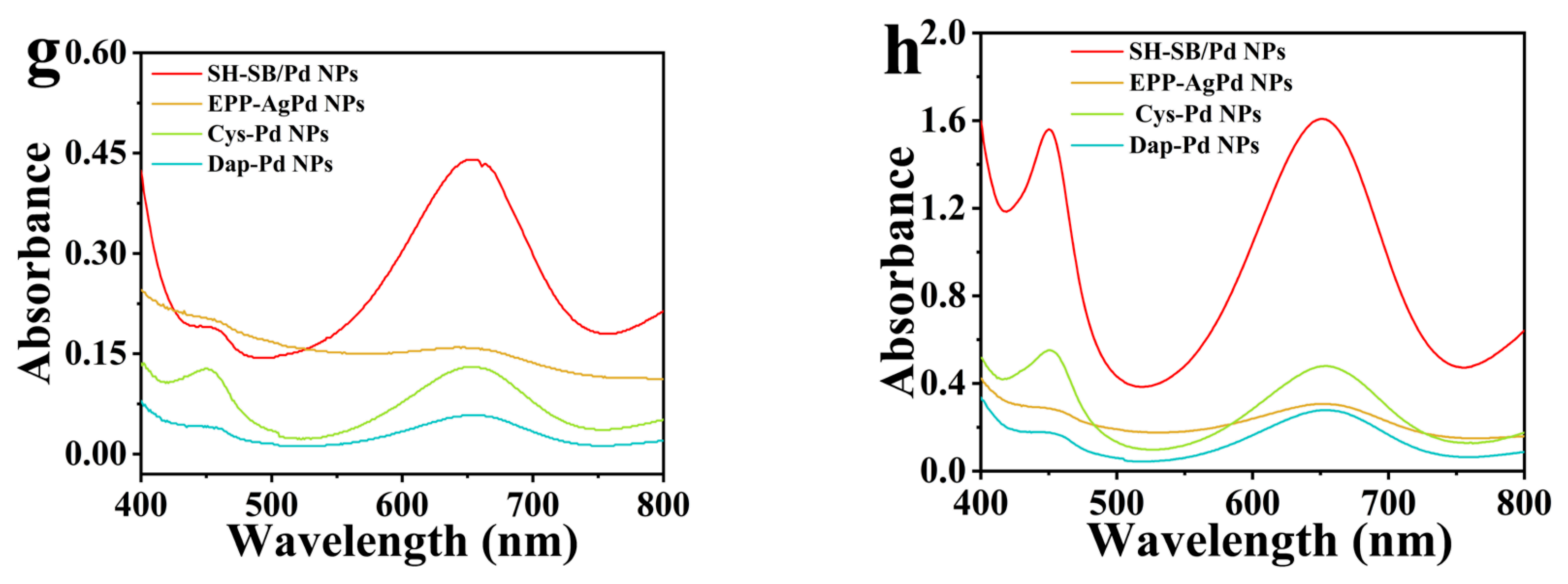
Enzyme-like activities of SH-SB/Pd NPs with (**a**,**b**) TMB, (**c**,**d**) OPD and (**e**,**f**) ABTS as substrates, respectively, and (**g**,**h**) comparison of enzyme-like activities of different nanomaterials.

O_2_ or H_2_O_2_ was catalyzed by SH-SB/Pd NPs to produce ROS, which in turn catalyzed colorless TMB to produce blue oxTMB, and produced a distinct absorption peak at 652 nm. The oxidase-like activity of SH-SB/Pd NPs was investigated under the condition of 30 °C and pH = 4, as shown in Figure 3a. The absorbance at 652 nm of 1—TMB + SH-SB/Pd NPs was 0.44, which was much stronger than the 0.104 of 2—TMB + H_2_O_2_, indicating that SH-SB/Pd NPs had strong oxidase-like activity at 30 °C and pH = 4.

The absorbance of the group 1 - TMB + SH-SB/Pd NPs + H_2_O_2_ at 652 nm was 1.608 after the reaction, as shown in Figure 3b, which was 3.20 times and 15.46 times that of the TMB + SH-SB/Pd NPs group and the TMB + H_2_O_2_ group, respectively, indicating that SH-SB/Pd NPs not only had oxidase-like activity, but also had strong peroxidase-like activity. OPD and ABTS can also be used as substrates for colorimetric reactions, and the UV-Vis spectra after oxidation will show absorption peaks at 448 nm and 420 nm, respectively. In total, 5 mM OPD and 2.5 mM ABTS were used as colorimetric substrates in Figure 3c–f. The changes in their absorption peak intensity proved that SH-SB/Pd NPs had stronger catalytic activity than unstable Pd NPs.

In addition, EPP-AgPd NPs, Dap-Pd NPs, and Cys-Pd NPs were prepared using elm pod polysaccharide (EPP), organic small molecule daptomycin (Dap), and amphiphilic molecule cysteine (Cys) as stabilizers, respectively. According to Figure 3g,h, the oxidase-like activity and peroxidase-like activity of SH-SB/Pd NPs were significantly stronger than those of other nanozymes, possibly because the sulfhydryl groups in SH-SB combined with the Pd surface to form stable Pd-S bonds, regulating the Pd electronic structure and d-band center, and promoting the adsorption and activation of substrates (O_2_/H_2_O_2_). Amino groups and sulfonic acid groups endow the surface of SH-SB/Pd NPs with appropriate charges and strong hydrophilicity, facilitating the electrostatic enrichment of positively charged TMB substrates. Compared with EPP, Dap and Cys, the modification of SH-SB can expose more active sites, and amphiphilic groups of SH-SB/Pd NPs can participate in electron transfer, synergistically enhancing the catalytic efficiency of the enzyme-like reaction.

### 3.3. Environmental Effects and Catalytic Kinetics

**Figure 4:**
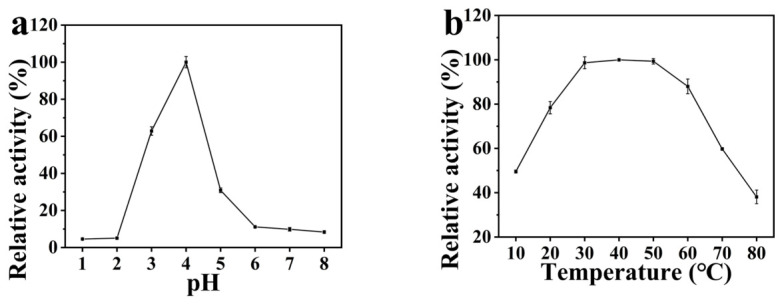

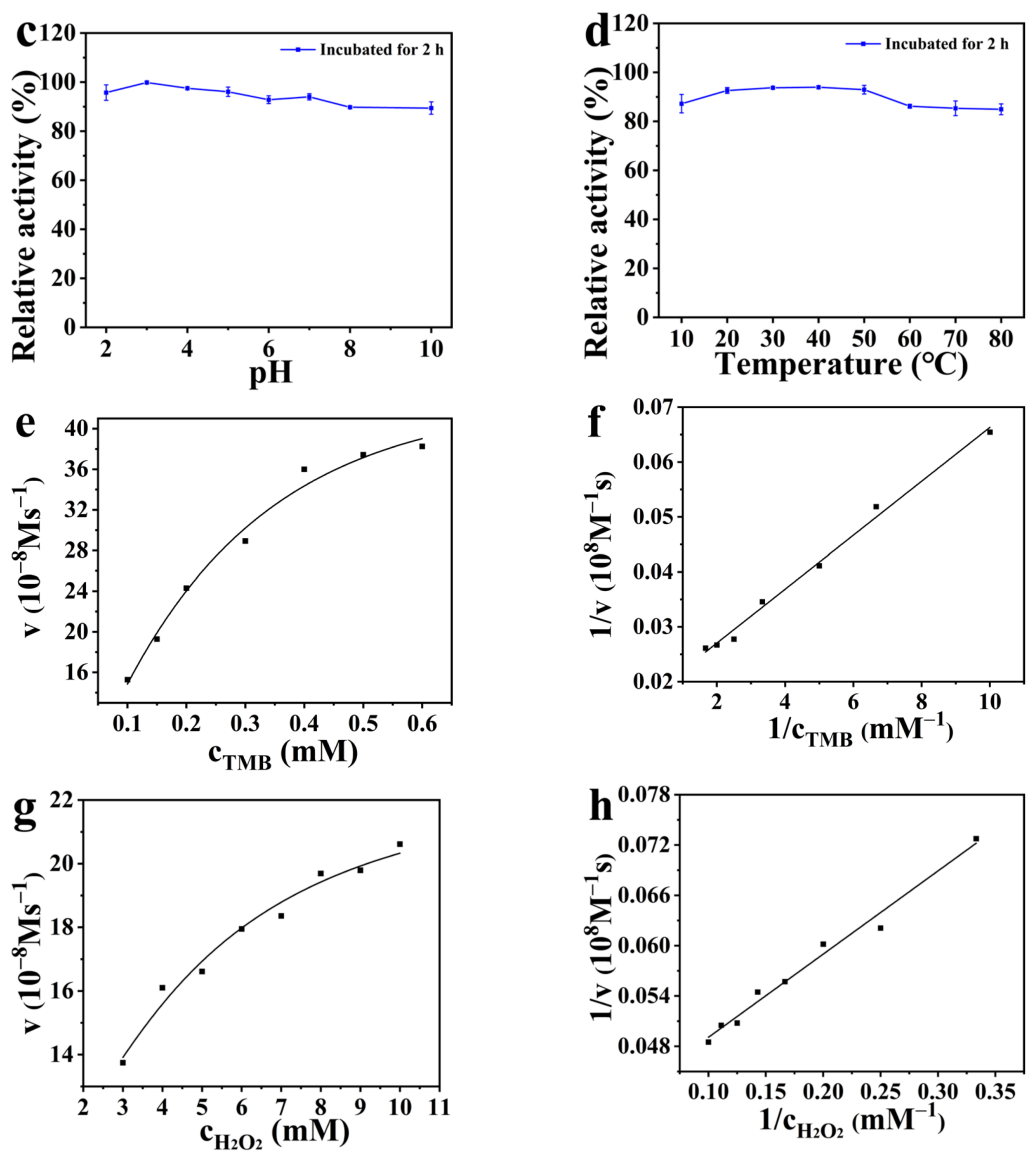
Effect of pH and temperature on (**a**,**b**) peroxidase-like activity, (**c**,**d**) stability of peroxidase-like activity of SH-SB/Pd NPs, the catalytic velocity curves of different concentrations of (**e**) H_2_O_2_ and (**f**) TMB as substrates and (**g**,**h**) the curves obtained by the double reciprocal plotting method.

Enzyme activity is greatly affected by environmental conditions, especially temperature and pH. SH-SB/Pd NPs exhibited the highest peroxidase-like activity towards TMB at pH = 4 and T = 40 °C. The peroxidase-like activity of SH-SB/Pd NPs remained basically unchanged at pH = 1–2, and gradually increased and decreased at pH = 2–8, with the strongest activity at pH = 4 in Figure 4a. SH-SB/Pd NPs maintained high peroxidase-like activity in the range of 20–60 °C, and the highest at 40 °C in Figure 4b. In Figure 4c,d, when SH-SB/Pd NPs were incubated at different pH (2–10) and temperature (10–80 °C) for 2 h, the relative peroxidase-like activity of SH-SB/Pd NPs was maintained at 89.41–99.84% and 84.93–93.92%, respectively. SH-SB/Pd NPs had good stability of enzyme-like activity under harsh pH and temperature.

In Figure 4e–h, TMB solution (0.1–0.6 mM) and H_2_O_2_ solution (1–10 mM) were used for the study of the catalytic kinetics of SH-SB/Pd NPs. The catalytic kinetic parameters of SH-SB/Pd NPS were calculated by double reciprocal mapping. *K*_*m*(*TMB*)_ = 0.28, *V*_*m*(*TMB*)_ = 58.04 × 10^−8^ Ms^−1^; *K*_*m*(*H*__2*O*__2__)_ = 2.53, *V*_*m*(*H*__2*O*2__)_ = 25.53 × 10^−8^ Ms^−1^. A small *K_m_* value indicates a high affinity of the enzyme for the reaction substrate [33]. Compared with the traditional HRP and other nanomaterials in Table 1, SH-SB/Pd NPs had a smaller *K_m_* when TMB as the substrate, indicating that SH-SB/Pd NPs had a stronger affinity for TMB. The special charge conduction structure of zwitterionic SH-SB enhanced the affinity of SH-SB/Pd NPs to TMB and accelerated their binding speed, which is conducive to the occurrence and acceleration of enzymatic reaction.

### 3.4. Catalytic Mechanism of SH-SB/Pd NPs

**Figure 5:**
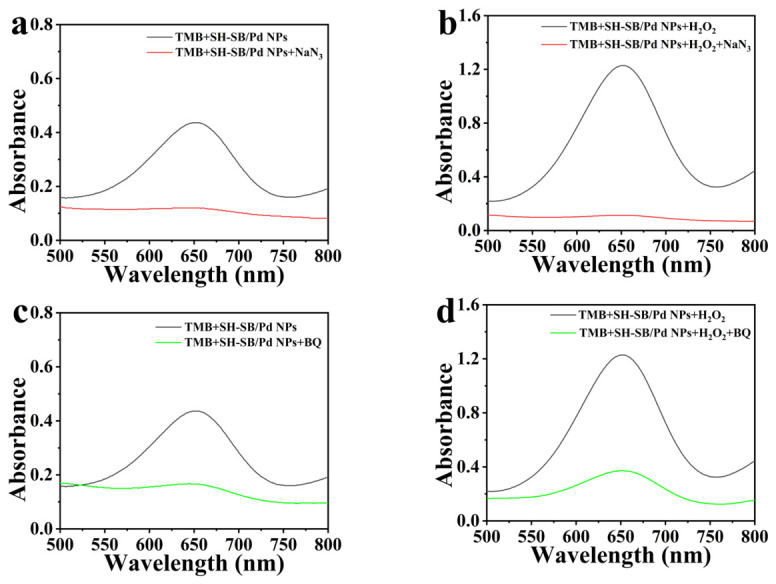

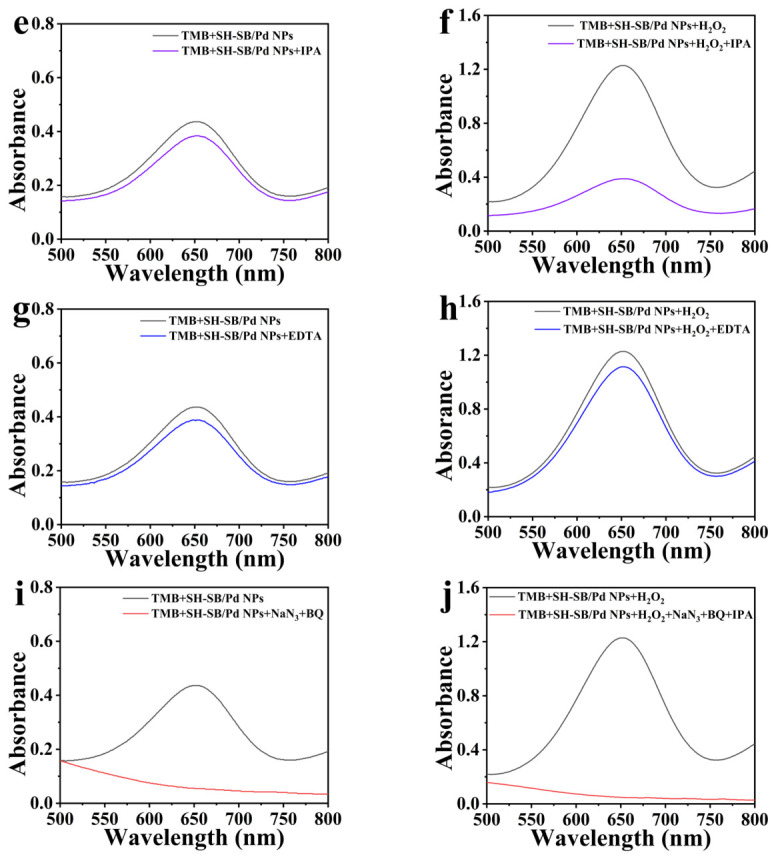
Effects of (**a**,**b**) NaN_3_, (**c**,**d**) BQ, (**e**,**f**) IPA, (**g**,**h**) EDTA (50 mM) and (**i**,**j**) NaN_3_ + BQ + IPA + EDTA as ROS inhibitors on the enzyme-like activity of SH-SB/Pd NPs with TMB as substrate.

NaN_3_ [41], BQ [42], EDTA [43] and IPA [44] solution (50 mM) were used as ^1^O_2_, O_2_^•−^, h^+^ and •OH inhibitors respectively to explore the ROS species generated during the reaction. As shown in Figure 5a,c, after NaN_3_ and BQ were added, the absorption peaks at 652 nm were significantly reduced compared with the control group (TMB + SH-SB/Pd NPs), indicating that NaN_3_ and BQ largely prevent the catalytic oxidation of TMB. In Figure 5e,g, the absorbance at 652 nm had little change after adding IPA and EDTA, indicating that EDTA and IPA had little effect on the oxidase-like activity of SH-SB/Pd NPs. When NaN_3_ and BQ were added at the same time in Figure 5i, there was no absorption peak at 652 nm, which proved that all ROS generated during the reaction were inhibited, indicating that the ROS generated during the oxidation of TMB by SH-SB/Pd NPs as an oxidase were ^1^O_2_ and O_2_^•−^.

The addition of NaN_3_, BQ and IPA significantly weakened the peroxidase-like activity of SH-SB/Pd NPs in Figure 5b,d,e. In Figure 5h, if EDTA was added, there was no significant effect on catalytic oxidation reaction of TMB. According to Figure 5j, after NaN_3_, BQ and IPA were added at the same time, the absorption peak of the mixed solution at 652 nm disappeared. These phenomena indicated that three ROS were produced during the catalytic oxidation of TMB, ^1^O_2_, O_2_^•−^ and •OH, when SH-SB/Pd NPs acted as a peroxidase.

### 3.5. GSH Biological Detection Platform

**Figure 6:**
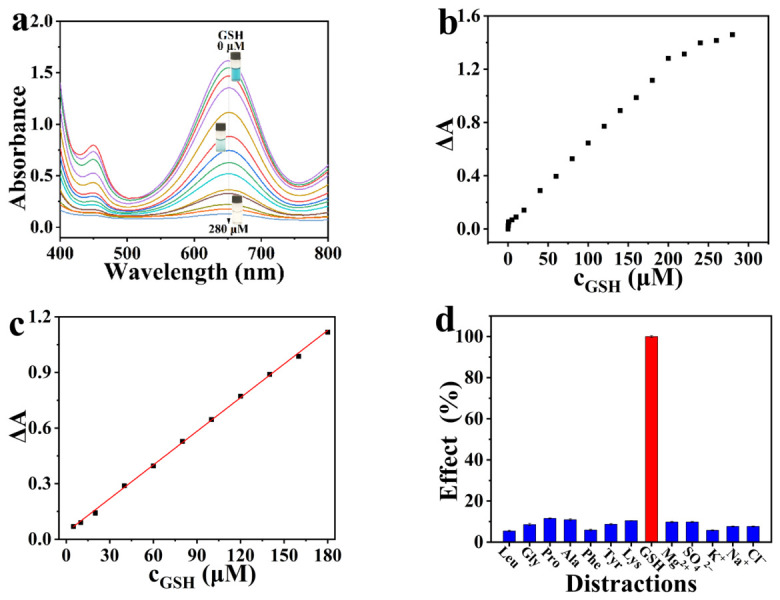
(**a**,**b**) Establishment and (**c**) anti-interference ability of GSH biological detection platform based on SH-SB/Pd NPs with TMB as substrate, and (**d**) the changes in the hydrodynamic size of SH-SB/Pd NPs after being incubated with fibrinogen solution.

The reducing power of GSH will reduce blue oxTMB to colorless TMB. Therefore, a biological detection platform for GSH was established based on SH-SB/Pd NPs using TMB as a colorimetric substrate. As shown in Figure 6a, with the increase in the concentration of GSH solution (0–280 μM), the absorbance at 652 nm decreased and the color faded gradually, indicating that more and more oxTMB was reduced. The standard curve of GSH biological detection platform (ΔA = 0.0378c_GSH_ + 0.006 (R^2^ = 0.999)) was shown in Figure 6b. As shown in Table 2, the detection range of this GSH biological detection platform was 0.135–180 μM, and LOD = 0.135 μM. The GSH biological detection platform had good sensitivity.

In addition, leucine, glycine, proline, phenylalanine, tyrosine, alanine, lysine and Mg^2+^, Na^+^, K^+^, SO_4_^2−^ and Cl^−^ were used as interfering substances [45] to test the anti-interference ability of the GSH biological detection platform, as shown in Figure 6c. GSH was replaced with interfering substances (molar ratio of interfering substances: GSH = 10:1). The response of the colorimetric system in the presence of GSH was set as 100% as a reference to normalize the data. The maximum influence of all interferences on the GSH biological detection platform was 11.58%, indicating that the GSH biological detection platform had good selectivity and reliability. The recovery rate of the GSH biological detection platform was measured by mice serum. As shown in Table 3, the recoveries of added samples were 100.23% and 100.12%, and the relative standard deviations (RSD) were 1.99% and 0.50%, respectively. This GSH biological detection platform is reliable.

**Table 2:** Comparison of different biosensors on GSH detection.

Nanomaterials	Detection Range (μM)	LOD (μM)	Ref.
SH-SB/Pd NPs	0.135–180	0.135	This work
MOF mimic enzyme (Fe/Cu = 1:1)	2–20	0.439	[46]
CoSe_2_ microspheres	0.005–10	4.62 × 10^−3^	[47]
Pd_150_-PCRPNPs	2–300	1.63	[48]
Co^2+^@XO	33–1300	13.7	[49]
CuS@CDs	5–300	0.0527	[50]

### 3.6. In Vitro Treatment of HeLa Cells

**Figure 7:**
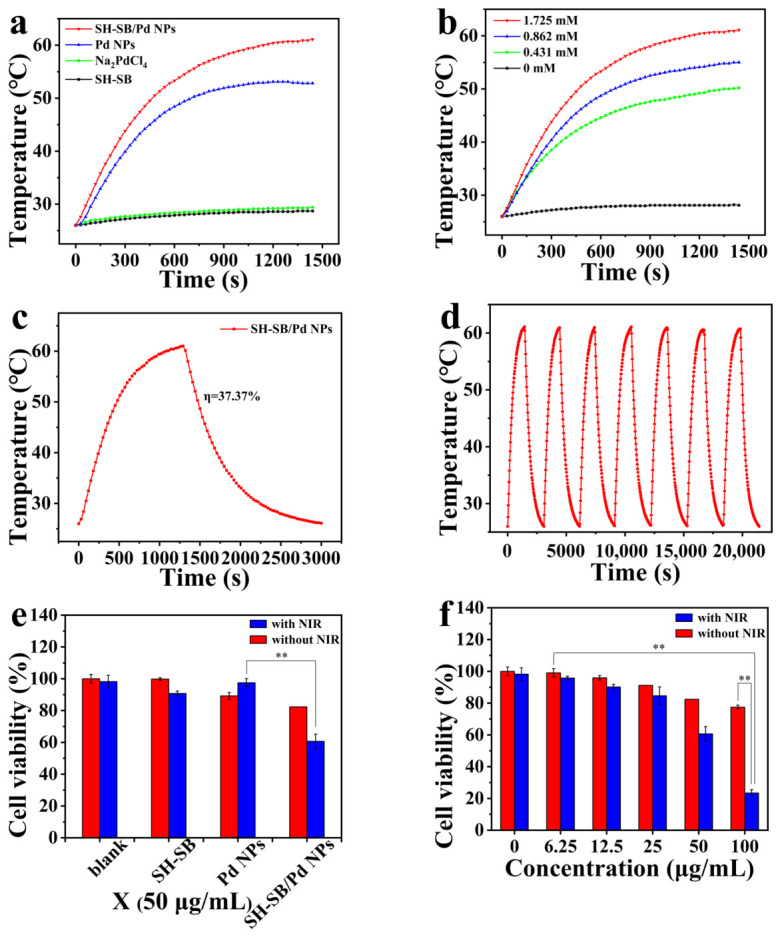
(**a**–**d**) Photothermal properties of SH-SB/Pd NPs and (**e**,**f**) the viability of HeLa cells treated with SH-SB/Pd NPs (** *p* < 0.01).

Figure 7a shows the temperature change in SH-SB/Pd NPs solution (C_Pd_ = 1.725 mM), SH-SB solution (5 mg/mL), Na_2_PdCl_4_ solution (2 mM) and Pd NPs solution (C_Pd_ = 1.725 mM) irradiated by the 808 nm laser for 1440 s. The temperature changes in SH-SB and Na_2_PdCl_4_ were very small. The ΔT_Pd NPs_ was 26.8 °C, and the ΔT_SH-SB/Pd NPs_ was 35.1 °C, which was 8.3 °C higher than Pd NPs, indicating that SH-SB as a stabilizer improved the photothermal properties of Pd NPs. As shown in Figure 7b, when the concentration of SH-SB/Pd NPs was 0.431 mM and 1.725 mM, T_SH-SB/Pd NPs_ was 50.2 °C and 61.1 °C, respectively. SH-SB/Pd NPs still had good photothermal properties at low concentrations. The photothermal ability enhanced with the increase in the concentration of SH-SB/Pd NPs and agglomeration did not occur due to the stability of the zwitterion SH-SB. As shown in Figure 7c, SH-SB/Pd NPs had a high photothermal efficiency, η = 37.37%. The photothermal properties of SH-SB/Pd NPs remained consistent in 7 cycles in Figure 7d indicated that the photothermal performance was not damaged by long-term, multiple laser exposures. Effective photothermal therapy depends on the good photothermal properties of SH-SB/Pd NPs.

As shown in Figure 7e, the viability of HeLa cells treated with 50 μg/mL Pd NPs solution, SH-SB solution and SH-SB/Pd NPs solution was 97.49%, 90.73% and 82.38%, respectively. SH-SB/Pd NPs killed more HeLa cells than Pd NPs. The cell viability of cell + SH-SB/Pd NPs + NIR group with additional 808 nm laser irradiation for 10 min was 60.64%. Under the condition of laser irradiation, the cytotoxicity of SH-SB/Pd NPs was further enhanced. The tumor inhibition ability of the cell + Pd NPs + NIR group was lower than that of cell + Pd NPs group, which may be due to the agglomeration of unstable Pd NPs due to photothermal effects, reducing the inhibitory capacity of HeLa cells.

With the increase in the concentration of SH-SB/Pd NPs in the range of 6.25–100 μg/mL, the inhibition of SH-SB/Pd NPs on HeLa cells enhanced gradually, as shown in Figure 7f. The viability of HeLa cells treated with 100 μg/mL SH-SB/Pd NPs + NIR was only 23.44% of the original. The thermal effect of SH-SB/Pd NPs resulted in the thermal ablation death of 76.56% of HeLa cells. The inhibitory capacity of SH-SB/Pd NPs on tumor cells was greatly enhanced with the aid of the 808 nm laser. SH-SB/Pd NPs + NIR effectively inhibited the proliferation of HeLa cells, indicating that SH-SB/Pd NPs + NIR had good tumor inhibition performance, and will have broad application prospects in cancer therapy.

## 4. Conclusions

In summary, uniformly dispersed SH-SB/Pd nanozymes (7.3 ± 2.4 nm) were successfully constructed using SH-SB, which endowed the nanoparticles with enhanced colloidal stability. Compared with conventional Pd-based nanozymes, the zwitterionic interface effectively regulated surface hydration and charge distribution, leading to good oxidase-like activity and peroxidase-like activity of SH-SB/Pd NPs. At pH = 4 and T = 40 °C, SH-SB/Pd NPs had the highest peroxidase-like activity, and the catalytic kinetics conforms to the traditional *Michaelis–Menten* equations. SH-SB/Pd NPs exhibited efficient oxidase-/peroxidase-mimicking behavior, enabling ROS generation via both H_2_O_2_-dependent and independent pathways. Benefiting from the high catalytic efficiency, a sensitive and reliable biological platform for GSH was established with a detection range of 0.135–180 μM and LOD = 0.135 μM. With the help of the 808 nm laser, the inhibition rate of SH-SB/Pd NPs (100 μg/mL) on HeLa cells was 76.56%, depending on the good photothermal properties of SH-SB/Pd NPs. The performance of SH-SB/Pd NPs in GSH detection and tumor inhibition in vitro suggests that SH-SB/Pd NPS will have broad application prospects in biomedicine. This work highlights zwitterionic surface engineering as an effective strategy to enhance the stability, catalytic performance, and biomedical applicability of nanozyme systems. Despite these advantages, further studies are still required to evaluate in vivo pharmacokinetics and catalytic behavior in complex biological microenvironments. Future studies could focus on extending this double-layer nanozyme strategy to other metal systems, optimizing tumor targeting efficiency, and integrating multimodal imaging and therapeutic functions to achieve precise biomedical applications.

## Figures and Tables

**Table 1:** Catalytic kinetic parameters of SH-SB/Pd NPs and some reported nanomaterials.

Catalysts	*K*_m_ (mM)		*V*_m_ (10^−8^ Ms^−1^)		References
	TMB	H_2_O_2_	TMB	H_2_O_2_	
SH-SB/Pd NPs	0.28	2.53	58.04	25.53	this work
HRP	0.434	3.70	10.00	8.71	[34]
Fe_3_O_4_/Ag Au	0.270	13.2	54.2	29.5	[35]
CuONRs@Pd_6_NPs	3.74	2.94	1.23	1.65	[36]
ZIF-67	13.69	19.9	31.96	1.92	[37]
CD/g-C_3_N_4_	0.35	5.76	2.67	3.80	[38]
MoS_2_	0.824	2.08	1.16	1.25	[39]
Cu NCs	1.125	2.52	7.20	12.8	[40]

**Table 3:** Determination of GSH in mice serum (*n* = 3).

Sample	Spiked GSH (μM)	Found GSH (μM)	Recovery (%)	RSD (%)
Serum 1	0 100	6.12 ± 0.16 100.23 ± 1.99	- 100.23	0.26 1.99
Serum 2	0 100	3.83 ± 0.054 100.12 ± 0.50	- 100.12	1.41 0.50

## Data Availability

The original contributions presented in this study are included in the article. Further inquiries can be directed to the corresponding author.

## Abbreviations

The following abbreviations are used in this manuscript:

SH-SB	Sulfhydryl sulfobetaine
NPs	Nanoparticles
GSH	Glutathione
ROS	Reactive oxygen species
LOD	Limit of detection
